# A nomogram based on nutritional status and A^2^DS^2^ score for predicting stroke-associated pneumonia in acute ischemic stroke patients with type 2 diabetes mellitus: A retrospective study

**DOI:** 10.3389/fnut.2022.1009041

**Published:** 2022-10-13

**Authors:** Xiaodong Song, Yang He, Jie Bai, Jun Zhang

**Affiliations:** ^1^Department of Neurology, Peking University People’s Hospital, Beijing, China; ^2^Department of Infectious Diseases, The First Affiliated Hospital of Chongqing Medical University, Chongqing, China

**Keywords:** stroke-associated pneumonia, nutritional status, A^2^DS^2^ score, acute ischemic stroke, type 2 diabetes mellitus

## Abstract

**Background:**

Stroke-associated pneumonia (SAP) commonly complicates acute ischemic stroke (AIS) and significantly worsens outcomes. Type 2 diabetes mellitus (T2DM) may contribute to malnutrition, impair innate immunity function, and increase the probability of SAP occurrence in AIS patients. We aimed to determine early predictors of SAP in AIS patients with T2DM and to construct a nomogram specifically for predicting SAP in this population by combining the A^2^DS^2^ score with available nutrition-related parameters.

**Methods:**

A total of 1,330 consecutive AIS patients with T2DM were retrospectively recruited. The patients were randomly allocated to the training (*n* = 887) and validation groups (*n* = 443). Univariate and multivariate binary logistic regression analyses were applied to determine the predictors of SAP in the training group. A nomogram was established according to the identified predictors. The areas under the receiver operating characteristic curve (AUROC) and calibration plots were performed to access the predictive values of the nomogram. The decision curve was applied to evaluate the net benefits of the nomogram.

**Results:**

The incidence of SAP was 9% and 9.7% in the training and validation groups, respectively. The results revealed that the A^2^DS^2^ score, stroke classification, Geriatric Nutritional Risk Index, hemoglobin, and fast blood glucose were independent predictors for SAP. A novel nomogram, A^2^DS^2^-Nutrition, was constructed based on these five predictors. The AUROC for A^2^DS^2^-Nutrition (0.820, 95% CI: 0.794–0.845) was higher than the A^2^DS^2^ score (0.691, 95% CI: 0.660–0.722) in the training group. Similarly, it showed a better predictive performance than the A^2^DS^2^ score [AUROC = 0.864 (95% CI: 0.828–0.894) vs. AUROC = 0.763 (95% CI: 0.720–0.801)] in the validation group. These results were well calibrated in the two groups. Moreover, the decision curve revealed that the A^2^DS^2^-Nutrition provided an additional net benefit to the AIS patients with T2DM compared to the A^2^DS^2^ score in both groups.

**Conclusion:**

The A^2^DS^2^ score, stroke classification, Geriatric Nutritional Risk Index, hemoglobin, and fast blood glucose were independent predictors for SAP in AIS patients with T2DM. Thus, the proposed A^2^DS^2^-Nutrition may be a simple and reliable prediction model for SAP occurrence in AIS patients with T2DM.

## Introduction

Stroke-associated pneumonia (SAP) is a spectrum of pneumonia complications that develop in non-ventilated patients following a stroke (1). The incidence of SAP was estimated to be 12.3% [95% confidential interval (CI), 11%–13.6%] (2). SAP significantly lengthens hospitalization and raises hospital costs (3–5). Furthermore, it is independently associated with worse functional outcomes and increased mortality rates (5, 6).

Diabetes was discovered to be related to SAP (7). Moreover, diabetes was found in approximately 33% of ischemic stroke patients and was closely associated with poor outcomes in this population (8). Patients with type 2 diabetes mellitus (T2DM) were more susceptible to community-acquired pneumonia (relative risk = 1.64, 95% CI, 1.55–1.73) than the non-T2DM in a meta-analysis incorporating about 15 million people (9). A plausible explanation is that hyperglycemia impair immunological function in T2DM patients (10). Furthermore, a significant malnutrition prevalence was found in hospitalized older patients with T2DM (11), due to gastroparesis, diet changes, and chronic kidney disease (12). Malnutrition is an issue commonly related to secondary immune deficiency and infection risk (13). Therefore, it is reasonable to pay more attention to the risk of SAP in acute ischemic stroke (AIS) patients with T2DM.

SAP is considered a potentially modifiable complication affecting stroke prognosis. Thus, identifying vulnerable patients is crucial to tailor monitoring and prophylactic strategies. Efforts have been made to establish prediction SAP scales, including the A^2^DS^2^, the PANTHERS, the ISAN, and the AIS-APS score (14). The A^2^DS^2^ score is the most extensively utilized risk scale in practice. It has had a moderate predive accuracy for SAP (AUROC = 0.85, 95% CI, 0.82–0.88) (15). In addition, some simple nutrition-related biomarkers, such as hyperglycemia (16) and serum album (17), had good predictive value for SAP. However, no existing research has been made to specifically predict SAP in patients with comorbidity of AIS and T2DM. There is also no prediction scale that takes nutritional risk status into account as an important factor.

In the present study, we retrospectively evaluated the relevance of the A^2^DS^2^ score with SAP in AIS patients with T2DM. Furthermore, we attempted to establish an individualized nomogram for discerning SAP in these patients by combining the A^2^DS^2^ score with simple nutrition-related biomarkers.

## Materials and methods

### Patients selection

This was a retrospective study conducted at the Peking University People’s Hospital (PKUPH) between January 2015 and September 2021. Consecutive AIS patients with T2DM who were admitted within 24 h of symptom onset were enrolled in our cohort. All participants met the WHO criteria for the diagnosis of AIS (18). The diagnosis of T2DM was based on the American Diabetes Association diagnostic criteria (19) or clear T2DM history. The exclusion criteria included the following: (1) age < 18 years; (2) diagnosis of transient ischemic attacks (TIA) or cerebral hemorrhage; (3) active pulmonary or other sites infection within the last 14 days; (4) history of severe hepatic diseases, hematological malignancy, or immunosuppressant treatment; (5) recent history of major trauma or surgery, and (6) incomplete medical records. This study was approved by the ethics committee at PKUPH and conducted in accordance with the Declaration of Helsinki.

### Data collection

The demographic and clinical data were recorded at the time of admission, including age, sex, height, weight, stroke classification, National Institutes of Health Stroke Scale (NIHSS) score at admission, hypertension, dyslipidemia hyperuricemia, atrial fibrillation, previous myocardial infarction, chronic heart failure, previous stroke, dementia, dysphasia, chronic obstructive pulmonary disease, chronic kidney disease, malignancy, smoking status, and drinking status. AIS was classified into four subtypes, including total anterior circulation infarct, partial anterior circulation infarct, posterior circulation infarct, and lacunar infarct (LACI), according to the Oxfordshire Community Stroke Project (OCSP) classification system (20). Body mass index (BMI) was defined as the weight (kg) divided by height square (m^2^). As previously reported, the A^2^DS^2^ score was determined by summing up the points of the corresponding risk factors: 3 for age ≥ 75 years, 1 for atrial fibrillation, 2 for dysphasia, 1 for male, 0 for NHISS 0–4, 3 for NHISS 5–15, and 5 for NHISS ≥ 16 (21). The following laboratory parameters were collected within 24 h of admission: hemoglobin, neutrophils, lymphocyte, platelet, fast blood glucose (FBG), glycosylated hemoglobin, serum creatinine, estimated glomerular filtration rate, serum album (ALB), total cholesterol, total glyceride, high- density lipoprotein, and low-density lipoprotein. Neutrophil to lymphocyte ratio (NLR) was defined as the neutrophil count (10^9^/L) divided by lymphocyte count (10^9^/L). The Geriatric Nutritional Risk Index (GNRI) formula was as follows: GNRI = [1.489 × ALB (g/L)] + [41.7 × current weight (kg)/ideal body weight (kg)] (22). Ideal weight was calculated based on the Lorentz equations: for men: ideal body weight = height (cm) – 100 – [height (cm) - 150]/4; for women: ideal body weight = height (cm) – 100 – [height (cm) - 150]/2. GNRI scores > 98, 92–98, 82–91, and < 82 were considered to indicate absent, low, moderate, and severe malnutrition risk, respectively (22).

### Outcome definition

SAP was defined as pneumonia complicating the first week after AIS onset in non-ventilated patients (1). In our study, clinically suspected SAP was diagnosed based on the modified Centers for Disease Control and Prevention criteria, according to the clinical symptoms and laboratory tests (23). Definite SAP was confirmed by typical chest X-ray (CXR) changes within 48 h of antibiotic initiation (1). However, only 36% of clinically suspected SAP manifested typical pneumonia findings on an initial CXR (1). Hence, in clinical practice, pulmonary computed tomography was undertaken within 48 h after consent following the suspicion of pneumonia, albeit with a with normal CXR appearance (24).

### Statistical analysis

IBM SPSS Statistics version 22.0 (IBM Corp.) and R project for Statistical Computer version 4.1.2 were applied for the statistical analyses. A 2-sided *P*-value < 0.05 was considered statistically significant. Continuous variables were presented as mean with the standard deviation if normally distributed and median with interquartile range otherwise. The *t*-test and Mann–Whitney *U* test were performed for comparison among the groups in continuous variables. The Fisher’s exact test and Pearson’s Chi-square test were employed to explore differences in the categorical variables.

Random number generator of SPSS was applied for random grouping of the participants. Two-thirds of the patients were randomly allocated to the training group to establish a nomogram, and the remaining were allocated to the validation group. Univariate logistic regression was applied to identify the potential variables associated with SAP. Then, the variables were included in the multivariate logistic regression when the *P*-value < 0.05. The results of multivariate regression were visualized as a forest plot. A nomogram was constructed based on the multivariate logistic regression. The nomogram was compared with the A^2^DS^2^ score by performing the area under the receiver operating characteristic curve (AUROC). The calibration plots were used to access the performance of the nomogram with 1,000 bootstraps resamples. Moreover, the decision curve was applied to evaluate the net benefits of the nomogram.

## Results

### Clinical characteristics of the study cohort

**FIGURE 1:**
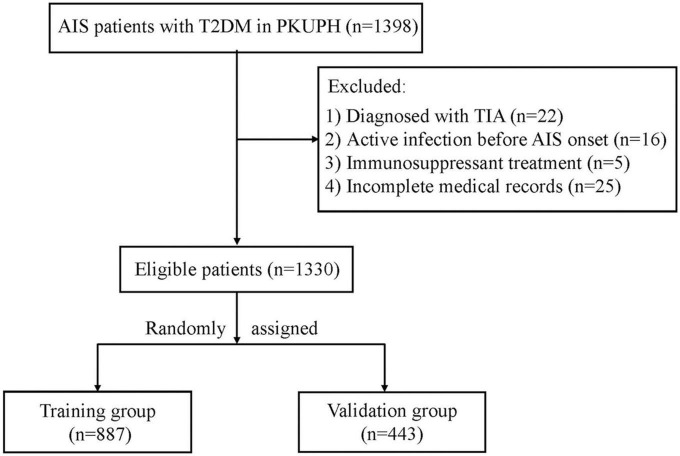
Study flow diagram. AIS, acute ischemic stroke; T2DM, type 2 diabetes mellitus; PKUPH, Peking University People’s Hospital; TIA, transient ischemic attack.

As shown in the flow chart of Figure 1, 1,398 T2DM patients with AIS attended PKUPH between January 2015 and September 2021. 22 patients diagnosed with TIA, 16 with the active infection before stroke onset (3 with sepsis and 13 with urinary tract infections), 5 receiving immunosuppressant treatment, and 25 with incomplete medical records were excluded from the cohort. Two-thirds of the patients (*n* = 887) were allocated to the training group, with the remaining (*n* = 443) allocated to the validation group. In addition to age, there was no significant difference in baseline demographic and clinical variables between the two groups (Table 1). SAP incidence rates were similar in both groups (9% vs. 9.7%, *P* = 0.683).

**TABLE 1:** Demographic and clinical variables of patients.

Variable	All cases (*n* = 1330)	Training Group (*n* = 887)	Validation Group (*n* = 443)	*P*-value
Male, n (%)	868 (65.3)	589 (66.4)	279 (63.0)	0.216
Age (years), mean (SD)	67.7 (12.0)	67.1 (11.9)	68.8 (12.2)	**0.016**
SAP, n (%)	123 (9.2)	80 (9.0)	43 (9.7)	0.683
BMI (kg/m^2^), mean (SD)	23.3 (4.6)	23.4 (4.6)	23.2 (4.7)	0.439
OCSP				
TACI, n (%)	105 (7.9)	70 (7.9)	35 (7.9)	0.995
PACI, n (%)	295 (22.2)	190 (21.4)	105 (23.7)	0.345
POCI, n (%)	261 (19.6)	177 (20.0)	84 (19.0)	0.667
LACI, n (%)	669 (50.3)	450 (50.7)	219 (49.4)	0.656
NIHSS at admission, median (IQR)	3.0 (2.0–5.0)	3.0 (2.0–5.0)	3.0 (2.0–5.0)	0.497
A^2^DS^2^, median (IQR)	1.0 (1.0–4.0)	1.0 (1.0–4.0)	1.0 (1.0–4.0)	0.913
GNRI, mean (SD)	100.6 (11.5)	100.7 (11.4)	100.3 (11.8)	0.522
Smoking, n (%)	511 (38.4)	354 (39.9)	157 (35.4)	0.114
Drinking, n (%)	322 (24.2)	210 (23.7)	112 (25.3)	0.519
Hypertension, n (%)	1,019 (78.4)	688 (77.6)	331 (74.7)	0.248
Dyslipidemia, n (%)	914 (68.7)	620 (69.9)	294 (66.3)	0.190
Hyperuricemia, n (%)	94 (7.1)	65 (7.3)	29 (6.5)	0.600
Atrial fibrillation, n (%)	97 (7.3)	63 (7.1)	34 (7.7)	0.705
Previous MI, n (%)	85 (6.4)	61 (6.9)	24 (5.4)	0.305
CHF, n (%)	16 (1.2)	8 (0.9)	8 (1.8)	0.154
Previous stroke, n (%)	112 (8.4)	76 (8.6)	36 (8.1)	0.785
COPD, n (%)	17 (12.8)	12 (1.4)	5 (1.1)	0.732
CKD, n (%)	45 (3.4)	33 (3.7)	12 (2.7)	0.336
Malignancy, n (%)	69 (5.2)	43 (4.8)	26 (5.9)	0.429
Dementia, n (%)	51 (3.8)	35 (3.9)	16 (3.6)	0.765
Dysphagia, n (%)	175 (13.2)	120 (13.5)	55 (12.4)	0.571
Hemoglobin (g/L), mean (SD)	133.4 (19.4)	133.7 (19.4)	132.8 (19.3)	0.413
Neutrophils (× 10^9^/L), median (IQR)	4.1 (3.3–5.4)	4.1 (3.3–5.4)	4.3 (3.4–5.4)	0.199
NLR, median (IQR)	2.5 (1.9–3.7)	2.5 (1.9–3.7)	2.7 (2.0–3.9)	0.087
Lymphocyte (× 10^9^/L), median (IQR)	1.6 (1.2–2)	1.6 (1.2–2.0)	1.6 (1.2–1.9)	0.517
Platelet (× 10^9^/L), median (IQR)	195.0 (159.3–232.0)	195.0 (159.0–232.0)	195.0 (161.0–232.0	0.729
FBG (mmol/L), median (IQR)	7.7 (6.2–10.3)	7.6 (6.2–10.4)	7.8 (6.2–9.8)	0.550
HbA1c (%), median (IQR)	7.7 (6.8–9.0)	7.7 (6.8–9.0)	7.6 (6.8–9.0)	0.832
SCR (μmol/L), median (IQR)	70.0 (58.3–86)	71.0 (59.0–87.0)	69.0 (58.0–86.0)	0.850
eGFR (ml/min*1.73m^2^), median (IQR)	88.3 (71.4–98.4)	88.6 (72.1–98.3)	87.4 (69.6–98.6)	0.313
ALB (g/L), median (IQR)	38.7 (35.9–40.9)	38.8 (36.0–40.9)	38.5 (35.6–41.1)	0.547
TC (mmol/L), median (IQR)	4.2 (3.4–5.1)	4.2 (3.4–5.1)	4.3 (3.5–5.2)	0.537
TG (mmol/L), median (IQR)	1.5 (1.1–2.1)	1.5 (1.2–2.1)	1.5 (1.1–2.0)	0.344
HDL (mmol/L), median (IQR)	1.0 (0.8–1.2)	1.0 (0.8–1.1)	1.0 (0.8–1.2)	0.720
LDL (mmol/L), median (IQR),	2.6 (2.0–3.3)	2.6 (2.0–3.3)	2.6 (2.0–3.4)	0.483

SAP, stroke-associated pneumonia; SD, standard deviation; IQR, interquartile range; BMI, body mass index, OCSP, Oxfordshire Community Stroke Project; TACI, total anterior circulation infarction; PACI, partial anterior circulation infarction; POCI, posterior circulation infarction; LACI, lacunar circulation infarction; NIHSS, National Institutes of Health Stroke Scale; GNRI, Geriatric Nutritional Risk Index; MI, myocardial infarction; CHF, chronic heart failure; COPD, chronic obstructive pulmonary disease; CKD, chronic kidney disease; NLR, neutrophil-to-lymphocyte ratio; FBG, fast blood glucose; HbA1c, glycosylated hemoglobin; SCR, serum creatinine clearance; eGFR, estimated glomerular filtration rate; ALB, album; TC, total cholesterol; TG, triglyceride; HDL, high-density lipoprotein cholesterol; LDL, low-density lipoprotein cholesterol.

Bold values mean *P*-value < 0.05.

### Baseline characteristics of patients in the training group stratified by SAP

Patients with SAP (*n* = 80) were older (71.3 ± 12.2 vs. 66.7 ± 11.8 years) and had higher NHISS [5.5 (3.0–11.3) vs. 3.0 (2.0–5.0)], A^2^DS^2^ scores [4.0 (1.0–6.0) vs. 1.0 (1.0–4.0)], and GNRI (91.7 ± 11.4 vs. 101.6 ± 11.0) than those without SAP (*n* = 807). Patients diagnosed with SAP showed lower BMI (21.1 ± 3.5 vs. 23.6 ± 4.6 kg/m^2^) and had higher incidence of non-LACI (76.3% vs. 46.6%), atrial fibrillation (13.8% vs. 6.4%), previous myocardial infarction (16.3% vs. 5.9%), and dysphagia (11.6% vs. 32.5%) than its counterparts. For laboratory features, the levels of hemoglobin (117.9 ± 29.0 vs. 135.3 ± 17.4 g/L), lymphocyte [1.1 (0.7–1.5) vs. 1.6 (1.2–2.0) × 10^9^/L], and ALB [35.3 (31.8–38.2) vs. 39.0 (36.5–41.0) g/L] were lower in the SAP group than in the non-SAP group. In addition, the levels of neutrophils [5.3 (3.8–7.0) vs. 4.0 (3.2–5.1) × 10^9^/L], NLR [4.4 (2.0–6.9) vs. 2.5 (1.9–3.5) × 10^9^/L], and FBG [9.8 (8.0–15.6) vs. 7.4 (6.1–10.0) × 10^9^/L] were higher in the SAP group when compared with its counterparts (Table 2).

**TABLE 2:** Univariate regression analysis of SAP in the training group.

Variable	Non-SAP group (*N* = 807)	SAP group (*N* = 80)	*P*-value	OR (95% CI)	*P*-value
Male, n (%)	540 (66.9)	49 (61.3)	0.306	0.782 (0.4870–1.254)	0.307
Age (years), mean (SD)	66.7 (11.8)	71.3 (12.2)	**0.001**	1.035 (1.014–1.056)	**0.001**
BMI (kg/m^2^), mean (SD)	23.6 (4.6)	21.1 (3.5)	**< 0.001**	0.857 (0.804–0.911)	**< 0.001**
OCSP					
LACI, n (%)	431 (53.4)	19 (23.8)		Reference	
Non-LACI, n (%)	376 (46.6)	61 (76.3)	**< 0.001**	3.680 (2.159–6.272)	**< 0.001**
NIHSS at admission, median (IQR)	3.0 (2.0–5.0)	5.5 (3.0–11.3)	**< 0.001**	1.072 (1.045–1.099)	**< 0.001**
A^2^DS^2^, median (IQR)	1.0 (1.0–4.0)	4.0 (1.0–6.0)	**< 0.001**	1.368 (1.242–1.507)	**< 0.001**
GNRI, mean (SD)	101.6 (11.0)	91.7 (11.4)	**< 0.001**	0.919 (0.897–0.940)	**< 0.001**
Absent malnutrition risk, n (%)	524 (64.9)	24 (30.0)		Reference	
Low malnutrition risk, n (%)	138 (17.1)	17 (21.3)		2.812 (1.462–5.410)	**0.002**
Moderate malnutrition risk, n (%)	117 (14.5)	25 (31.3)		4.877 (2.675–8.893)	**< 0.001**
High malnutrition risk, n (%)	27 (3.3)	15 (18.8)		12.681 (5.950–27.029)	**< 0.001**
Smoking, n (%)	323 (40.0)	31 (38.8)	0.824	0.948 (0.592–1.519)	0.824
Drinking, n (%)	190 (23.5)	20 (25.0)	0.770	1.083 (0.636–1.842)	0.770
Hypertension, n (%)	628 (77.8)	60 (75.0)	0.564	0.855 (0.502–1.457)	0.565
Dyslipidemia, n (%)	560 (69.4)	60 (75.0)	0.297	1.323 (0.781–2.243)	0.298
Hyperuricemia, n (%)	61 (7.6)	4 (5.0)	0.402	0.644 (0.228–1.819)	0.406
Atrial fibrillation, n (%)	52 (6.4)	11 (13.8)	**0.015**	2.315 (1.155–4.644)	**0.018**
Previous MI, n (%)	48 (5.9)	13 (16.3)	**0.001**	3.068 (1.583–5.947)	**< 0.001**
CHF, n (%)	7 (0.9)	1 (1.3)	0.730	1.447 (0.176–11.91)	0.731
Previous stroke, n (%)	70 (8.7)	6 (7.5)	0.720	0.854 (0.359–2.032)	0.721
COPD, n (%)	9 (1.1)	3 (3.8)	0.086	3.455 (0.916–13.028)	0.067
CKD, n (%)	27 (3.3)	6 (7.5)	0.110	2.342 (0.937–5.855)	0.069
Malignancy, n (%)	36 (4.4)	7 (8.8)	0.099	2.054 (0.883–4.778)	0.095
Dementia, n (%)	30 (3.7)	5 (6.3)	0.235	1.727 (0.651–4.582)	0.273
Dysphagia, n (%)	94 (11.6)	26 (32.5)	**< 0.001**	3.652 (2.182–6.112)	**< 0.001**
Hemoglobin (g/L), mean (SD)	135.3 (17.4)	117.9 (29.0)	**< 0.001**	0.963 (0.953–0.973)	**< 0.001**
Neutrophils (× 10^9^/L), median (IQR)	4.0 (3.2–5.1)	5.3 (3.8–7.0)	**< 0.001**	1.137 (1.052–1.229)	**0.001**
NLR, median (IQR)	2.5 (1.9–3.5)	4.4 (2.0–6.9)	**< 0.001**	1.089 (1.047–1.132)	**< 0.001**
Lymphocyte (× 10^9^/L), median (IQR)	1.6 (1.2–2.0)	1.1 (0.7–1.5)	**< 0.001**	0.293 (0.189–0.454)	**< 0.001**
Platelet (× 10^9^/L), median (IQR)	195.0 (160.0–229.0)	192.0 (129.8–241.8)	0.390	0.999 (0.996–1.002)	0.5732
FBG (mmol/L), median (IQR)	7.4 (6.1–10.0)	9.8 (8.0–15.6)	**< 0.001**	1.098 (1.048–1.151)	**< 0.001**
HbA1c (%), median (IQR)	7.6 (6.8–9.0)	8.0 (6.9–10.2)	0.148	1.122 (0.997–1.262)	0.057
SCR (μmol/L), median (IQR)	71.0 (60.0–86.0)	73.0 (47.8–101.3)	0.677	1.001 (0.998–1.003)	0.506
eGFR (ml/min*1.73m^2^), median (IQR)	88.8 (73.8–98.2)	86.8 (63.0–104.3)	0.467	0.993 (0.984–1.003)	0.175
ALB (g/L), median (IQR)	39.0 (36.5–41)	35.3 (31.8–38.2)	**< 0.001**	0.861 (0.822–0.902)	**< 0.001**
TC (mmol/L), median (IQR)	4.2 (3.4–5.1)	4.4 (3.0–5.5)	0.939	0.964 (0.818–1.136)	0.659
TG (mmol/L), median (IQR)	1.5 (1.2–2.1)	1.4 (1.2–2.2)	0.834	1.010 (0.887–1.150)	0.883
HDL (mmol/L), median (IQR)	1.0 (0.8–1.2)	1.0 (0.7–1.1)	0.243	0.605 (0.240–1.529)	0.228
LDL (mmol/L), median (IQR),	2.6 (2.0–3.3)	2.8 (1.7–3.6)	0.889	0.985 (0.787–1.234)	0.898

SAP, stroke-associated pneumonia; OR, odds ratios; SD, standard deviation; IQR, interquartile range; BMI, body mass index, OCSP, Oxfordshire Community Stroke Project; LACI, lacunar circulation infarction; NIHSS, National Institutes of Health Stroke Scale; GNRI, Geriatric Nutritional Risk Index; MI, myocardial infarction; CHF, chronic heart failure; COPD, chronic obstructive pulmonary disease; CKD, chronic kidney disease; NLR, neutrophil-to-lymphocyte ratio; FBG, fast blood glucose; HbA1c, glycosylated hemoglobin; SCR, serum creatinine clearance; eGFR, estimated glomerular filtration rate; ALB, album; TC, total cholesterol; TG, triglyceride; HDL, high-density lipoprotein cholesterol; LDL, low-density lipoprotein cholesterol.

Bold values mean *P*-value < 0.05.

### Development of a predictive nomogram for SAP

**FIGURE 2:**
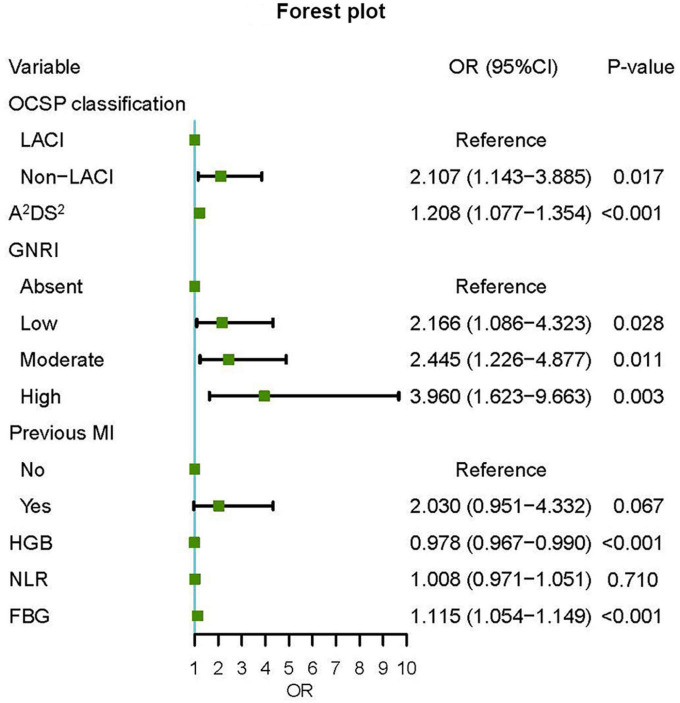
Forest plot of odds ratios for SAP. An OR (odds ratios) > 1 meant an increased risk of SAP (stroke-associated pneumonia); an OR < 1 meant the opposite. OCSP, Oxfordshire Community Stroke Project; LACI, lacunar circulation infarction; GNRI, Geriatric Nutritional Risk Index; MI, myocardial infarction; NLR, neutrophil-to-lymphocyte ratio; FBG, fast blood glucose.

**FIGURE 3:**
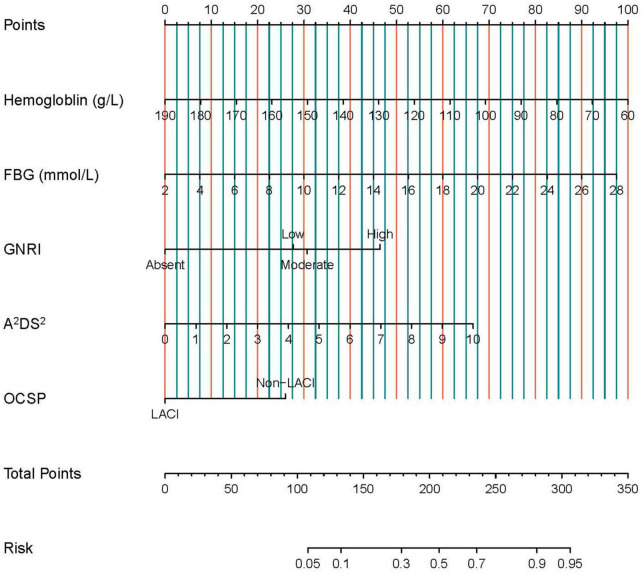
The nomogram for predicting the occurrence of SAP in AIS patients with T2DM. Each risk factor is given a point based on the nomogram. The total points are calculated by summing up the corresponding scores on the points scales of each variable. The probability of SAP can be obtained by locating the total points on the “Total Points” axis and drawing a vertical line down to the “SAP Risk” axis. SAP, stroke-associated pneumonia; GNRI, Geriatric Nutritional Risk Index; OCSP, Oxfordshire Community Stroke Project; LACI, lacunar circulation infarction; FBG, fast blood glucose.

According to the univariate logistic regression analysis, fifteen factors were significantly correlated with SAP. They were age, BMI, OCSP classification, NIHSS score, A^2^DS^2^ score, Geriatric Nutritional Risk Index (GNRI), atrial fibrillation, previous myocardial infarction, dysphagia, hemoglobin, neutrophils, lymphocyte, NLR, FBG, and ALB (Table 2). Age, atrial fibrillation, dysphagia, and NIHSS were not included in the multivariate regression analysis since they were parts of the A^2^DS^2^ score. In addition, neutrophils and lymphocytes, and BMI and ALB, were excluded from subsequent analysis because they were the main elements of the NLR and GNRI formula separately. Finally, the remaining seven factors were incorporated into the multivariate logistic regression. The results revealed that A^2^DS^2^ score, OCSP classification (non-LACI), GNRI, hemoglobin, and FBG were independent predictors for SAP (Figure 2). All VIF values were ≤ 1.15, indicating low collinearity among the variables. Based on these independent predictors, a nomogram, A^2^DS^2^-Nutrition, was constructed to predict the probability of SAP in AIS patients with T2DM (Figure 3). The A^2^DS^2^-Nutrition assigned a probability (5%–95%) of SAP occurrence. Each risk factor was assigned a point based on the nomogram. The total points were calculated by adding the corresponding scores on each variable’s scale point. The probability of SAP could be obtained by locating the total points on the “Total Points” axis and drawing a vertical line down to the “SAP Risk” axis. For example, the A^2^DS^2^-Nutrition assigns a 50% probability of SAP in a LACI patient (0 points) with A^2^DS^2^ = 6 (40 points), glucose = 14 mmol/L (45 points), low malnutrition risk (27.5 points), and hemoglobin = 70 g/L (92.5 points), with a total score of 205 points.

### Internal and external validation of the nomogram

**FIGURE 4:**
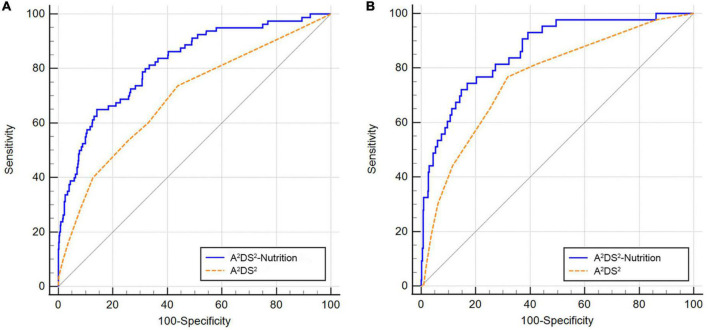
Comparison of the AUROC values between the A^2^DS^2^-Nutrition and A^2^DS^2^ score. **(A)** In the training group, the A^2^DS^2^-Nutrition had a larger AUROC than the A^2^DS^2^ [0.820 (95% CI, 0.794–0.845) vs. 0.691 (95% CI, 0.660–0.722), *P* < 0.001]; **(B)** In the validation group, the AUROC of A^2^DS^2^-Nutrition was larger than the A^2^DS^2^ [0.864 (95% CI, 0.828–0.894) vs. 0.763 (95% CI, 0.720–0.801), *P* = 0.002].

**FIGURE 5:**
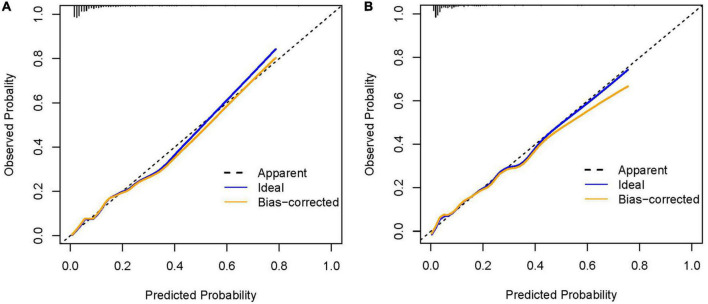
The calibration curve of the A^2^DS^2^-Nutrition. **(A)** Calibration curve in the training group; **(B)** Calibration curve in the validation group.

**FIGURE 6:**
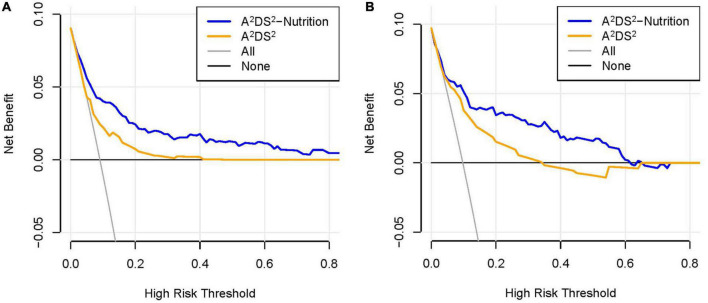
Comparison of the decision curves between the A^2^DS^2^-Nutrition and A^2^DS^2^ score. **(A)** The decision curve in the training group; **(B)** the decision curve in the validation group.

The AUROC for this nomogram in the training and validation groups was 0.820 (95% CI: 0.794–0.845) and 0.864 (95% CI: 0.828–0.894), respectively, which were significantly higher than those for A^2^DS^2^ [trainings group, 0.691 (95% CI: 0.660–0.722); validation group, 0.763 (95% CI: 0.720–0.801)] (Figures 4A,B). In the training group, the calibration plot for SAP probability showed good accordance between the predicted probability and the actual observation (Figure 5A); the mean absolute error was 0.009. Similarly, the calibration plot of the observed against the predicted possibility of SAP demonstrated satisfactory concordance in the validation group; the mean absolute error was 0.015 (Figure 5B). In addition, for a threshold probability range of 3% to 97%, using the A^2^DS^2^-Nutrition would yield a greater net benefit than applying the A^2^DS^2^ in the training group (Figure 6A). This means that our nomogram is superior to the A^2^DS^2^ sore for treatment decisions (e.g., prophylactic antibiotic therapy) in such a wide range of pre-defined risk thresholds. For a threshold probability range of 2% to 66%, the A^2^DS^2^-Nutrition would provide more net benefit to AIS patients with T2DM than the A^2^DS^2^ in the validation group (Figure 6B).

## Discussion

The A^2^DS^2^ score, stroke classification (non-LACI), GNRI, hemoglobin, and FBG, were independent predictors of SAP in this research. A nomogram was established according to these factors for predicting SAP risk in AIS patients with T2DM. It would assist neurologists in identifying vulnerable patients, making early decisions, and tailoring preventive strategies.

SAP is usually ascribed to stroke-induced immunodepression (5, 6). In addition, pneumonia is more frequent in T2DM patients since hyperglycemia predominantly affects innate immunity (10, 25). Moreover, diabetes is a critical factor for malnutrition in stroke patients [odds ratio (OR) = 2.60, 95% CI: 2.24–3.03] (26), resulting in compromised immunity and increased infection rates (13). Diabetes may increase the risk of pneumonia in people suffering from an ischemic stroke (27, 28). Furthermore, T2DM patients always have poorer clinical outcomes after infection, as evidenced by increased hospitalization incidence, length of stay, and complications (29). Nevertheless, no clinical tool exists to explicitly predict SAP occurrence in AIS patients with T2DM. Although the SAP scoring systems, such as A^2^DS^2^, have been established for stroke, they have not been specifically validated in the T2DM population. In the present study, the A^2^DS^2^ score had an insufficient accuracy in predicting SAP occurrence in the training group [AUROC = 0.691 (95% CI: 0.660–0.722)]. Furthermore, our nomogram had a higher AUROC for discriminating SAP compared with the A^2^DS^2^ score. This suggests that the A^2^DS^2^-Nutrition has a more reliable prediction ability. More importantly, the nomogram assesses the individual risk based on the A^2^DS^2^ score and nutritional status, which are easily accessible on admission.

The A^2^DS^2^ score was constructed using data from a large cohort of 15,335 stroke patients in Germany. This research revealed that the score had a high sensitivity (83%) and specificity (72%) (21). The A^2^DS^2^ score considers age, male sex, atrial fibrillation, dysphagia, and NIHSS, all of which are the mainly elements in other well-recognized SAP scales (30, 31). However, other research reported inconsistencies in A^2^DS^2^ sensitivities and specificities, probably due to different designs and study populations (15). Therefore, we modified the A^2^DS^2^ score by in our cohort combining it with stroke classification and nutritional parameters. The GNRI score is a simple scale system initially designed to assess malnutrition risk among elderly patients (22). Subsequently, the GNRI score was applied to predict sepsis mortality (32) and the probability of surgical site infections (33, 34). Malnutrition risk was found in 5.3% of 8,698 Chinese patients with AIS according to the GNRI score (35). In contrast, 22.1% (294/1330) of our cohort had moderate or severe malnutrition risk defined by GNRI score. A possible reason may be that T2DM patients often have an improper diet due to poor diabetes-related nutrition knowledge and barriers to dietary adherence (36). In line with this, the malnutrition risk of older COVID-19 patients with diabetes was 2.12 times higher than in those without diabetes in China (37). Lower GNRI scores were associated with an increased risk of long-term death and significant disability in patients with AIS (35). In addition, the GNRI score was employed as a biomarker for SAP risk classification (38). Patients with SAP showed lower GNRI scores than without SAP (96.88 ± 9.36 vs. 100.88 ± 8.25), indicating its predictive values in AIS patients (38). Considering the high malnutrition risk rate in elderly diabetic patients (11), the GNRI score was included in our prediction model as a critical element.

Hyperglycemia significantly contributed to SAP occurrence in our cohort. This is evidenced by the results of several studies, which demonstrated that glucose level has satisfactory values for predicting SAP (16, 39). Noteworthy, hyperglycemia reduces the neutrophil’s bactericidal ability by inhibiting migration, phagocytosis, and superoxide production (40). Furthermore, it may increase the circulation cytokine concentrations, such as tumor necrosis factor-α and interleukin-6, significantly contributing to post-stroke infection (16). In our prediction model, decreased hemoglobin was another independent factor for SAP. Anemia is evaluated to be present in 30% of AIS patients (41). Anemia on admission not only increased the mortality rate (OR = 1.97, 95% CI 1.57–2.47) (41), but also contributed to in-hospital pneumonia incidence (OR = 1.71, 95% CI 1.35–2.17) in stroke patients (42). A reason might be that the anemic patients may be nutritionally imbalanced and immune-compromised. In our study, decreased hemoglobin possibly indirectly reflected the malnutritional risk status of the AIS patients with T2DM.

To our best knowledge, A^2^DS^2^-Nutrition is the first nomogram for specifically predicting SAP in AIS patients with T2DM based on routine data. SAP is notorious for increased care dependency, 30-day mortality, and 1-year mortality (5, 6). Hence, our prediction model may facilitate physicians in making individualized decisions, and implementing early empirical antibiotic therapy, thereby benefiting patients in the long run. Furthermore, the nomogram highlights the need for glycemic control and nutritional support for T2DM patients. A recent study has shown that almost 50% of the patients were non-adherent to their T2DM treatment regimens (43). Thus, hyperglycemia should be strictly monitored and controlled upon admission. Meanwhile, timely nutritional supplements for malnourished individuals may reduce SAP risk.

There remain some limitations in this study. First, to make the nomogram more user-friendly, we did not include infecting-related data (e.g., C-reactive protein and procalcitonin), which were routinely unavailable upon admission in this research. This may affect the accuracy of the prediction model. Second, all the patients were from the same PKUPH center, and the nomogram needs external cohorts to be validated. Finally, our prediction model was developed in the Chinese cohort, limiting the extrapolation of the findings to regions outside our country. However, the model was entirely based on available data, providing the foreign researchers with a new applicable way in the future. Hence, multicenter studies with large sample sizes are required to corroborate the findings of our study.

## Conclusion

The A^2^DS^2^ score, stroke classification, Geriatric Nutritional Risk Index, hemoglobin, and fast blood glucose were independent predictors for SAP in AIS patients with T2DM. The A^2^DS^2^-Nutrition might be a simple and reliable tool for predicting SAP occurrence in this population. It may assist physicians in identifying high-risk patients, making individual decisions, and initiating prophylactic therapies early, thus benefiting the patients in the long run.

## Data availability statement

The original contributions presented in this study are included in the article/supplementary material, further inquiries can be directed to the corresponding author/s.

## Ethics statement

The studies involving human participants were reviewed and approved by the Ethics Committee at Peking University People’s Hospital. The ethics committee waived the requirement of written informed consent for participation.

## Author contributions

JZ and JB designed the study and interpreted the results of this work. YH and XS participated in the data collection. XS and JB analyzed the data and wrote the manuscript. All authors contributed to the discussion and approved the submitted version of the manuscript.

## Abbreviations

SAP: stroke-associated pneumonia
AIS: acute ischemic stroke
T2DM: type 2 diabetes mellitus
AUROC: areas under the receiver operating characteristic curve
CI: confidence interval
OR: odds ratio
PKUPH: Peking University People’s Hospital
TIA: transient ischemic attack
NIHSS: National Institutes of Health Stroke Scale
LACI: lacunar infarct
OCSP: Oxfordshire Community Stroke Project
BMI: body mass index
FBG: fast blood glucose
ALB: album
NLR: neutrophil to lymphocyte ratio
GNRI: Geriatric Nutritional Risk Index
CXR: chest X-ray.
